# A retrospective analysis of *Pseudomonas aeruginosa* bloodstream infections: prevalence, risk factors, and outcome in carbapenem-susceptible and -non-susceptible infections

**DOI:** 10.1186/s13756-019-0520-8

**Published:** 2019-04-25

**Authors:** Qingyi Shi, Chen Huang, Tingting Xiao, Zhenzhu Wu, Yonghong Xiao

**Affiliations:** 1 State Key Laboratory for Diagnosis and Treatment of Infectious Disease, Collaborative Innovation Center for Diagnosis and Treatment of Infectious Diseases, the First Affiliated Hospital, College of Medicine, Zhejiang University, Hangzhou, People’s Republic of China; 2 Department of Respiratory medicine, Ningbo Medical Treatment Center Li huili Hospital, Ningbo, People’s Republic of China

**Keywords:** Carbapenem-non-susceptible *Pseudomonas aeruginosa*, Bloodstream infection, Clinical characteristic, Risk factor, Prognosis

## Abstract

**Background:**

*Pseudomonas aeruginosa* (PA) is a leading cause of nosocomial infections, and carbapenem non-susceptible strains are a major threat to patient safety.

**Methods:**

A single center, retrospective comparative analysis of carbapenem-non-susceptible PA (CnSPA) and carbapenem-susceptible PA (CSPA) bloodstream infections (BSIs) was conducted between January 1, 2007, and December 31, 2016. Prevalence and risk factors associated with CnSPA BSIs were examined.

**Results:**

The study enrolled 340 patients with PA BSIs; 30.0% (*N* = 101) of patients had CnSPA. High APACHE II scores (≥15), central venous catheterization, and delayed application of appropriate definitive therapy were independently associated with higher risk of mortality in PA BSIs. Multivariate analysis revealed that respiratory disease and exposure to carbapenems within the previous 90 days to onset of BSI were independent risk factors for acquisition of CnSPA BSIs. Overall all-cause 30-day mortality associated with PA BSIs was 26.8% (91/340). In addition, mortality was higher in patients with CnSPA than in those with CSPA (37.6% vs. 22.2%, respectively; *P* = 0.003). Corticosteroid therapy and delayed receipt of effective definitive therapy were independent risk factors for death from CnSPA BSIs.

**Conclusion:**

Increased incidence of CnSPA BSIs was observed during the study period, with higher mortality seen in patients with these infections. Respiratory disease and exposure to carbapenems were independent risk factors for development of CnSPA BSIs. Appropriate definitive therapy reduced mortality rates. BLBLIs were as effective as carbapenems as a treatment for PA BSIs.

**Electronic supplementary material:**

The online version of this article (10.1186/s13756-019-0520-8) contains supplementary material, which is available to authorized users.

## Introduction

*Pseudomonas aeruginosa* (PA) is responsible for 10–15% of nosocomial infections worldwide [1]. Infections caused by PA isolates are typically difficult to treat due to intrinsic antibiotic resistance and a remarkable ability to acquire resistance to multiple groups of antimicrobial agents [2]. Such infections are associated with high mortality rates, which range from 18 to 61% [3].

Carbapenems are regarded as a drug of last resort and are used to treat severe infections caused by multidrug-resistant PA. However, increasing use of carbapenems has increased the prevalence of carbapenem-non-susceptible (CnS) PA strains. Moreover, such strains are often resistant to other drugs such as β-lactams and quinolones [4]. According to surveillance from the United States and Europe, the prevalence of CnSPA increased from 4% in the 1990s to 14–36% in the 2000s [5, 6]. In China, the prevalence of CnSPA is > 30% [5]. Thus, CnSPA has caused many nosocomial outbreaks [7–9].

Previous studies show that inappropriate antimicrobial therapy and/or delayed initiation of effective antimicrobial therapy is associated with an adverse outcome [4, 10]. However, it is unclear whether combination antimicrobial therapy plays a role in mortality associated with PA bloodstream infections (BSIs) [11–14]. Appropriate combination therapy has a favorable effect on survival of patients with febrile neutropenia [15]; however, few studies have focused on antimicrobial therapy. Treatment of infections caused by CnSPA remains a notable challenge in the clinical setting.

Here, we aimed to identify risk factors for CnSPA BSIs and evaluate clinical treatment responses and factors associated with mortality in patients with PA BSIs or CnSPA BSIs.

## Materials and methods

### Study cohort

This retrospective cohort study was carried out at The First Affiliated Hospital of Zhejiang University, a 2500-bed tertiary teaching hospital for adults in Zhejiang, China. The study focused on episodes of PA BSI occurring between January 1, 2007, and December 31, 2016. Episodes of BSI were identified from the clinical microbiology laboratory database. Patient data (demographics and clinical and microbiological data) were retrieved from patient charts. The inclusion criteria were as follows: (i) patients aged ≥18 years; (ii) the first episode of PA BSI occurred during the study period; and (iii) patients met the diagnostic criteria for BSI [16]. In briefly, BSI was defined as the presence of viable bacteria in the bloodstream and clinical signs or symptoms response to infection. Outpatients or patients with incomplete data were excluded.

### Data collection

Demographic and clinical and microbiological data were retrieved from the electronic medical records system. The following data were collected: age; gender; underlying disease according to a crude Charlson Comorbidity Index (CCI) [17]; duration of hospital stay before and after BSI; duration of intensive care unit (ICU) stay before BSI; antimicrobial drug exposure; use of corticosteroids or other immunosuppressive agents within the 90 days prior to onset of BSI; history of an invasive procedure or surgery within the 90 days prior to onset of BSI; presence of neutropenia and severity of illness (estimated using the acute physiology and chronic health status scoring system II [APACHE II] and Pitt score); clinical and laboratory findings; and treatment and outcome.

### Definitions

The probable source of BSI was determined according to the definitions for nosocomial infections document published by the Centers for Disease Control [16]. Primary BSI was recorded if no source was identified. Neutropenia was defined as an absolute neutrophil count < 1500/μl at the time of BSI onset. Corticosteroid therapy was defined as administration of > 20 mg/day prednisone (or its equivalent) for a period of ≥7 days. Antimicrobial drug exposure was defined as use of antibiotics for more than 48 h within the 90 days prior to onset of BSI. Empirical therapy included all antimicrobial drugs administered to treat a suspected BSI. Definitive therapy included all anti-PA therapies administered within 7 days of receiving susceptibility testing results. Antimicrobial therapy was considered “appropriate” when the antimicrobial regimen included administration of at least one effective drug (as defined by the in vitro susceptibility testing results) during the infection episode, or “inappropriate” if this criterion was not met. Aminoglycoside monotherapy was defined as inappropriate therapy. During the study period, cefoperazone/sulbactam (administered at a ratio of 1:1) was used to treat PA BSI. The dose was classified as low (2.0–4.0 g/d) or high dose (6.0–8.0 g/d) [18]. The dose of piperacillin/tazobactam was classified as low (4 g/500 mg every 12 h), medium (4 g/500 mg every 8 h), or high (4 g/500 mg every 6 h) [19].All-cause in-hospital mortality included all causes of death during hospitalization.

Antimicrobial susceptibility of PA was determined using the Vitek2 system. The guidelines of the Clinical and Laboratory Standards Institute standards (2017) define carbapenem non-susceptibility as a minimum inhibitory concentration of ≥4 μg/ml imipenem or meropenem [20].

### Statistical analysis

Continuous variables are expressed as the mean ± standard deviation (assessed using Student’s t test) or as the median (range) (evaluated using the Wilcoxon rank-sum test) when the distribution was not normal. Categorical variables are expressed as percentages and were analyzed using the Chi-squared test or two-tailed Fisher exact test as appropriate. The strength of associations was determined by calculating the odds ratio (OR) and 95% confidence intervals (CIs). Two-tailed tests were used to determine statistical significance. All-cause in-hospital mortality was examined by logistic regression analysis. A *P* value < 0.05 was considered statistically significant. All statistical analyses were performed using SPSS version 24.0. The survival distribution function was estimated using the Kaplan-Meier product limit method. Nonparametric (log-rank and Wilcoxon) tests were used to compare survival functions between groups.

## Results

### Study cohort

During the study period, there were 832 episodes of PA BSI (Additional file 1: Figure S1). Of these, 12 patients aged < 18 years, 340 duplicate patients, 52 outpatients, and 92 patients with incomplete clinical data were excluded from the analyses. Thus, 340 patients (225 male and 115 female) were included and the average age of the patients was 58 years. 101 episodes (30.0%) were caused by CnSPA and 239 (70.0%) by Carbapenem-susceptible *Pseudomonas aeruginosa* (CSPA) strains. Data for both groups are summarized in Table 1. The sensitivity of CnSPA to beta-lactam/beta-lactam inhibitor combinations (BLBLIs) reached 51.5%, whereas carbapenems showed limited antimicrobial activity against BLBLI-non-susceptible PA (32.4%, 23/71) (Additional file 4: Table S4). The mean length of hospital stay before onset of PA BSI was 20.4 days, and 41.8% of patients resided in the ICU within the 90 days prior to onset of BSI. At onset of PA BSI, most patients resided in the ICU or hematology department. The sensitivity of PA to BLBLIs was higher than that to carbapenems (78.8% vs. 70.3%, respectively; *P* = 0.011). Over the past 10 years, the incidence of PA BSI has fluctuated from 0.027/1000 patient-days to 0.062/1000 patient-days, with the highest incidence occurring in the ICU (1.85/1000 patient-days); this proportion neither rose nor declined year on year (*P* = 0.180) (Additional file 2: Figure S2). Before PA BSI, 20.9% of thoracic or abdominal drainage fluid or sputum samples were positive for PA, whereas 25.6% patients had a BSI caused by other pathogens. The respiratory tract (35%) was the most common probable source of infection, followed by the biliary tract (16.5%). The annual incidence of CnSPA BSI increased year on year (*P* = 0.003; Additional file 3: Figure S3). The 30 day all-cause in-hospital mortality rate was 26.8% (91/340), and mortality in the CnSPA group was higher than that in the CSPA group (37.6% [38/101] vs. 22.2% [53/239], respectively; *P* = 0.003). The overall 30-day attributable mortality rate was 21.5% (73/340). The 30-day attributable mortality rates were similar among patients infected with CnSPA (26.7%, 27/101) or CSPA (19.2%, 46/239) (*P* = 0.125).

**Table 1:**
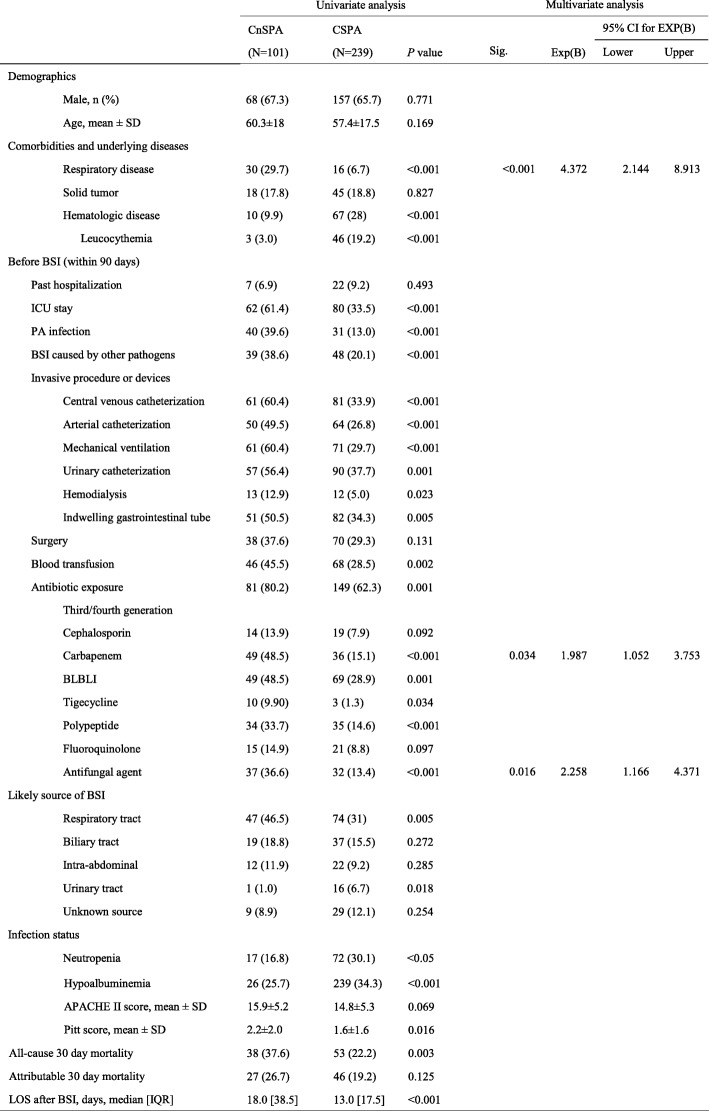
Demographics, clinical characteristics, infection status, and outcomes of patients with PA BSI

### Risk factors for acquisition of CnSPA BSIs

Univariate logistic analysis of patients with CSPA or CnSPA BSI (Table 1) revealed that patients with CnSPA tended to have respiratory disease (excluding lung cancer) or leucocythemia, prior to hospitalization in the ICU (*P* < 0.001), or to have undergone hemodialysis (*P* < 0.05), blood transfusion (*P* < 0.05), or a nonsurgical invasive procedure (including deep vein catheterization, arterial catheterization, mechanical ventilation, endotracheal intubation or incision, an indwelling urethral catheter, or an indwelling gastrointestinal tube; all *P* < 0.005). In addition, they were more likely to have respiratory tract infection (most probable infectious source), higher Pitt scores, and longer hospitalization days after BSI than those with CSPA. There was no significant difference between the groups in terms of APACHE II scores at onset of BSI. Also, patients with CnSPA were more likely to have been exposed to antibiotics during the 90 days prior to BSI onset (*P* = 0.001).

Multivariate logistic regression analysis (Table 1) identified the following as independent risk factors for CnSPA BSIs: an underlying respiratory disease (excluding lung cancer) (OR, 4.372; 95% CI, 2.144–8.913; *P* < 0.001), exposure to carbapenems in the 90 days prior to onset of BSI (OR, 1.987; 95% CI, 1.052–3.753; *P* = 0.034), and exposure to antifungal agents in the 90 days prior to onset of BSI (OR, 2.258; 95% CI, 1.166–4.371; *P* = 0.016).

### Risk factors for 30-day mortality in patients with PA BSIs

Of the 340 patients enrolled, 249 (73.2%) were classified as survivors and 91 (26.8%) as non-survivors. Univariate logistic analysis (Table 2) revealed that, compared with survivors, non-survivors had the following characteristics: respiratory disease (excluding lung cancer), a high APACHE II score on admission, prior hospitalization in the ICU within the 90 days before onset of PA BSI, and a longer ICU stay. In addition, it was more likely that respiratory tract infection was the probable infectious source or that they had undergone hemodialysis, blood transfusion, or a nonsurgical invasive procedure (including deep vein catheterization, arterial catheterization, mechanical ventilation, endotracheal intubation or incision, an indwelling urethral catheter, or an indwelling gastrointestinal tube). They were also more likely to have been exposed to corticosteroids and antibiotics (e.g., carbapenems, BLBLIs, linezolid, fosfomycin, and fluoroquinolone) and antifungal drugs during the 90 days prior to onset of BSI, to have higher Pitt scores (2.8 ± 2.5 vs. 1.4 ± 1.3, respectively; *P* < 0.001), to have a high APACHE II score (17.5 ± 5.8 vs. 14.8 ± 4.9, respectively; *P* < 0.001), and to have hypoalbuminemia. The rate of carbapenem non-susceptibility in non-survivors was higher than that in survivors.

**Table 2:**
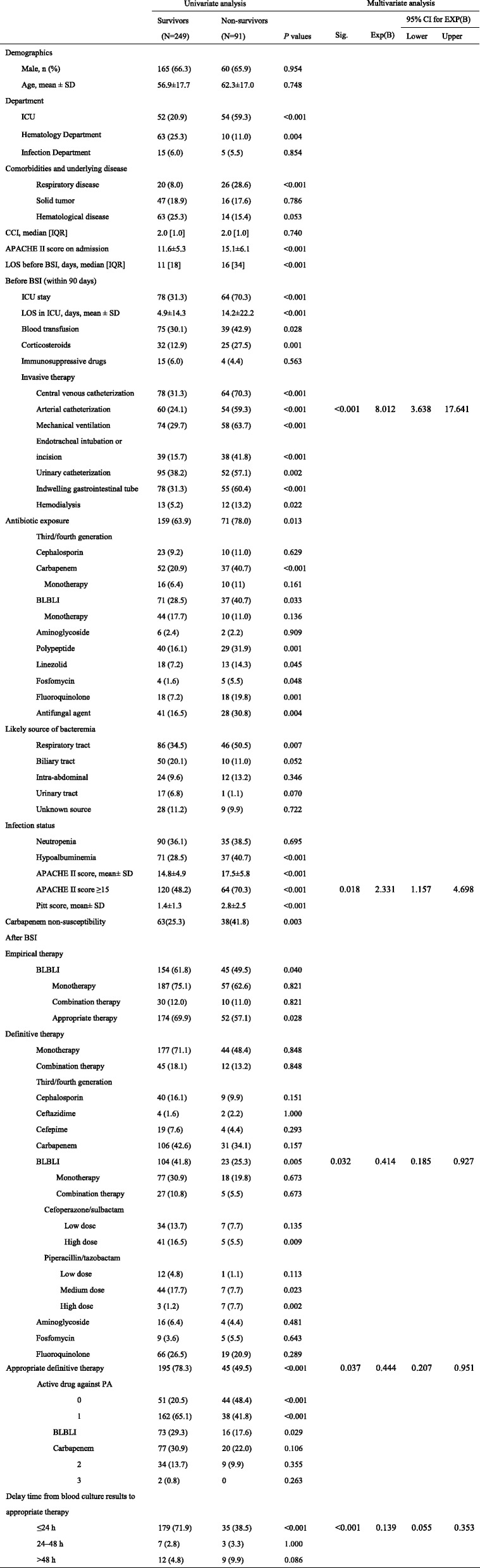
Risk factors associated with 30 day mortality in patients with PA BSIs

Univariate analysis (Table 2) revealed that combination therapy was no better than monotherapy at reducing mortality in patients with PA BSIs, which was similar for CSPA BSIs (Additional file 4: Table S2). And infection status was similar between patients received monotherapy and patients received combination therapy (Additional file 4: Table S3). Appropriate therapy (either empirical or definitive therapy) led to a significant reduction in the mortality rate of patients with PA BSIs. Definitive therapy with BLBLIs or carbapenems was associated with improved prognosis (survival rate, 81.9% (104/127) and 77.4% (106/137), respectively). Appropriate monotherapy with BLBLIs or carbapenems also improved the outcome of PA BSIs (survival rate, 82.0% (73/89) and 79.4% (77/97), respectively). In addition, receipt of prompt appropriate therapy, especially within 24 h (the interval between obtaining blood culture results and receipt of appropriate therapy) improved prognosis.

**Fig. 1:**
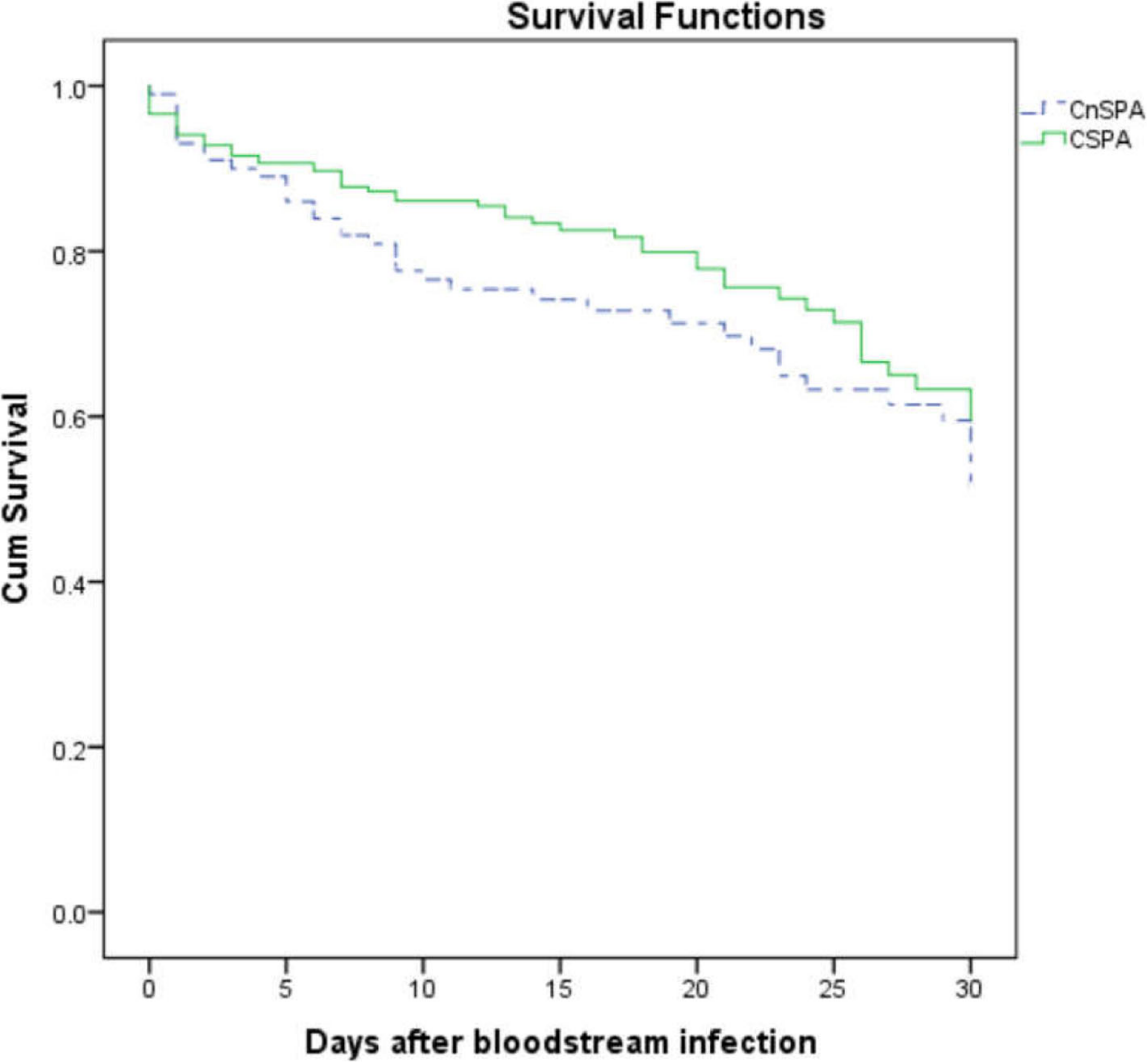
Kaplan-Meier survival estimates: patients with bloodstream infection caused by carbapenem non-susceptible (CnS) and carbapenem-susceptible (CS) *Pseudomonas aeruginosa* (PA) (*P* = 0.143)

Multivariate logistic regression analysis (Table 2) identified the following as independent risk factors for mortality in patients with PA BSIs: prior arterial catheterization before BSI (OR, 8.012; 95% CI, 3.638–17.641; *P* < 0.001) and APACHE II score ≥ 15 points (OR, 2.331; 95% CI, 1.157–4.698; *P* = 0.018). Independent factors that reduced the risk of mortality were appropriate definitive therapy (OR, 0.444; 95% CI, 0.207–0.951; *P* = 0.037), definitive therapy with BLBLIs (OR, 0.414; 95% CI, 0.185–0.927; *P* = 0.032), and receipt of prompt appropriate therapy (within 24 h) (OR, 0.139; 95% CI, 0.055–0.353; *P* < 0.001). The 30-day survival curve analysis revealed no significant difference in mortality between the CnSPA and CSPA groups (*P* = 0.143) (Fig. 1).

### Risk factors for 30-day mortality in patients with CnSPA BSIs

Of the 101 patients with CnSPA BSIs, 63 (62.4%) were classified as survivors and 38 (37.6%) as non-survivors. The 30-day mortality rate was 37.6%, and the average hospitalization time was 54 days. Univariate analysis showed that, compared with survivors (Additional file 4: Table S1), non-survivors had the following characteristics: respiratory disease (excluding lung cancer) or hypertension, prior ICU hospitalization, prior exposure to corticosteroids or BLBLIs, previous hemodialysis, a nonsurgical invasive procedure (including deep vein catheterization, arterial catheterization, mechanical ventilation, endotracheal intubation or incision, or an indwelling gastrointestinal tube), high APACHE II scores on admission (15.1 ± 6.2 vs. 11.9 ± 5.5, respectively; *P* = 0.009), a respiratory tract infection as the probable infectious source, hypoalbuminemia, and a high APACHE II score at onset of BSI (16.2 ± 5.9 vs. 13.9 ± 4.8, respectively; *P* = 0.036). Multivariate logistic regression analysis (Additional file 4: Table S1) identified prior exposure to corticosteroids as an independent risk factor for mortality in patients with CnSPA BSIs (OR, 8.055; 95% CI, 1.246–52.091, *P* = 0.028). Receipt of prompt appropriate definitive therapy (within ≤24 h) improved survival (OR, 0.108; 95% CI, 0.016–0.723; *P* = 0.022).

Of the 101 patients with CnSPA BSIs, 81 (80.2%) received empirical therapy and 30 (30.0%) received appropriate empirical therapy. In addition, 65 (64.4%) patients received empirical monotherapy and 16 (15.8%) received combination therapy. There was no significant difference in mortality between those receiving empirical monotherapy and those receiving empirical combination therapy (30.8% vs. 43.7%, respectively; *P* = 0.324). Finally, 80 (79.2%) patients received definitive treatment; of these, 55 received monotherapy, although there was no significant difference in mortality between those receiving definitive monotherapy and those receiving definitive combination therapy (29.1% vs. 28.0%, respectively; *P* = 0.920). Fifty-one patients (50.5%) received appropriate definitive therapy. Forty-one (40.6%) patients received one active drug, and 10 (9.9%) patients received at least two active drugs. There was no significant difference in mortality between those receiving appropriate definitive therapy and those receiving inappropriate definitive therapy (29.4% vs. 46.0%, respectively; *P* = 0.085) (Additional file 4: Table S1).

## Discussion

PA can cause life-threatening infections in hospitalized patients with severe underlying diseases or immunosuppression. Here, we found that, during the 90 days before onset, 41.8 and 25.6% patients were admitted to the ICU due to BSI caused by PA or BSI caused by other pathogens, respectively. Moreover, the majority of isolates were obtained from the ICU or hematology department. This is because patients in these settings are at particularly high risk of infection due to prolonged hospitalization, invasive medical procedures, and long-term use of antibiotics. Similar results were reported in a retrospective cross-sectional study by Lila, which identified PA as the most common pathogen recovered in the ICU [21]. Here, we found that the respiratory tract was the most common source of pathogens, followed by the biliary tract and an unknown source. Other clinical studies report that the major source of PA BSIs is the urinary tract [3, 22] or an unknown source [23]. As the outcome may be affected by the source of infection, recognizing a high risk of infection in a timely and accurate (i.e., abdominal infection, lower respiratory tract infection, or untargeted infection) manner can result in a positive outcome [24, 25].

Hospitalized patients often have a record of prior exposure to antibiotics or immunological system defects (e.g., prior exposure to antibiotics was noted in 80.2% of CnSPA vs. 60.9% of CSPA cases; *P* = 0.001). Exposure to antibiotics, especially monotherapy, can cause drug resistance (and even multidrug resistance) due to production of antibiotic-inactivating enzymes, low permeability of the bacterial outer membrane, overexpression of various efflux pumps, and loss of the outer membrane protein [4]. Once multidrug-resistant PA becomes established, few drugs are effective. Here, we showed that prior exposure to carbapenems or antifungals was a risk factor for acquisition of CnSPA BSIs. This may be because use of carbapenems not only promotes colonization of CnSPA and emergence of endogenous infection, but also triggers generation of CnSPA, in the medical environment [26]. The high prevalence of CnSPA colonization and the presence of CnSPA in the environment is associated with crossover spread of CnSPA in critically ill patients [27]. In addition, patients with prior exposure to antifungals often receive broad-spectrum antibiotics to treat severe infections [28]. We found that, compared with patients without a history of antifungal exposure, a higher proportion of patients with previous exposure to antifungals also received carbapenems (14.0% vs. 68.1%, respectively; *P* < 0.001). Moreover, patients exposed to antifungals are more likely to have received antineoplastic and immunosuppressive agents, broad-spectrum antibiotics, prosthetic devices and grafts, and more aggressive surgery [28], which may result in exacerbation of their condition and admission to the ICU. Of note, univariate analysis, not multivariate analysis, revealed a significant difference of BSI caused by other pathogens between CnSPA and CSPA (39,38.6% vs 48,20.1%, < 0.001). Antibiotic, especially carbapenems (71.8%,28/39) was used in CnSPA group before the onset of PA BSI.

According to surveillance from the United States and Europe, the prevalence of CnSPA increased from 4% in the 1990s to 14–36% in the 2000s [5, 6]. In China, the prevalence of CnSPA is > 30% [5]. The fact that the annual incidence of CnSPA BSI increased year on year represented an emerging challenge to public health. Carbapenem resistance in PA strains results from multiple mechanisms, including production of carbapenemases, overexpression of the efflux pump, or loss of outer membrane porins plus production of extended spectrum b-lactamase or AmpC b-lactamase [29].And in China, carbapenem resistance was mainly driven by the mutational inactivation of the *oprD* gene in the previous molecular epidemiology studies [30]. Skurnik et al. found that transposon insertions in the *oprD* gene led not only to carbapenem resistance but also to a dramatic increase in mucosal colonization and dissemination to the spleen in infected murine hosts [31]. It may resulted to the high mortality of PA BSI caused by CnSPA. Further studies should be focused on the relationship between the mechanism of carbapenem resistance and mortality in infected patients. It may be helpful to reveal the evolution of antimicrobial resistance and fitness in PA.

We found that the 30-day all-cause mortality rate for the CnSPA BSI group was 37.6%. However, mortality remained as high as 29.4% even when patients received appropriate definitive therapy. The all-cause and infection-attributable mortality rates in the CnSPA BSI group were higher than those in the CSPA BSI group. However, an association between carbapenem resistance and prognosis remains unclear [25, 32]. Previous studies show that infections caused by CnSPA result in higher mortality than those caused by susceptible strains [24]. A prospective multicenter study conducted in 2012 suggested that the higher Charlson index scores were, the less effect on mortality caused by carbapenem resistance. In other words, the association between carbapenem resistance and prognosis becomes less clear when other complications are factored in [33]. Furthermore, Lin et al. found that carbapenem resistance did not play a role in increasing mortality [34]. Thus, larger, well-designed prospective clinical trials are needed to evaluate the effect of carbapenem resistance on mortality.

There is no consensus regarding treatment of PA BSIs [13, 35]. A retrospective study shows that the efficacy of piperacillin-tazobactam is not inferior to that of other antibacterial agents, including carbapenems, when the chosen regimen of drug was sensitive against pathogen in vitro [36]. Here, we found that carbapenems and BLBLIs had similar effects on the outcome of PA BSIs (survival rate: 81.9% vs. 77.4%, respectively; *P* = 0.363). Moreover, subgroup analysis of appropriate monotherapy revealed that survival rates after treatment with BLBLIs and carbapenems were still similar (82.0% vs. 79.4%, respectively). In addition, there was no significant difference in survival rates after definitive therapy with BLBLIs or carbapenems (85.3% vs. 80.0%, respectively; *P* = 0.333). As mentioned previously, inappropriate therapy is an independent risk factor for a poor prognosis [37]. The promising effects of BLBLIs on the prognosis of PA BSI may be due to the high sensitivity of PA. The sensitivity of PA to BLBLIs was higher than that to carbapenems. Indeed, the sensitivity of CnSPA to BLBLIs reached 51.5%, whereas carbapenems showed limited antimicrobial activity against BLBLI-non-susceptible PA. Another study found that BLBLIs showed a significant negative correlation with the incidence of CnSPA when Bonferroni corrections were used to correct for multiple comparisons [38]. Therefore, use of BLBLIs to treat PA BSI is of benefit as it improves the outcome and inhibits generation of CnSPA.

Previous studies found that appropriate therapy, no matter whether that be monotherapy or combination therapy, led to a significant reduction in mortality of patients with PA infections [39]. A meta-analysis found that combination therapy was not more effective than monotherapy for reducing mortality due to PA BSI^11^. Our own findings are consistent with the above results. Of note, Kim et al. found that appropriate combination therapy was associated with better outcomes than appropriate monotherapy for patients with febrile neutropenia [12]. However, combination therapy was not better for patients with diverse disease severities or with CnSPA or CSPA. Inappropriate empirical therapy and delayed receipt of effective therapy are independent risk factors for death due to PA BSIs [37, 40]. Combination therapy is recommended because it contributes to “appropriateness” of therapy [34]. However, we found that appropriate empirical and definitive monotherapy was achieved in 85.7% (209/244) and 90.5% (200/221) of cases, respectively, which is close to the rates achieved by combination therapy. Moreover, our study found that the 30-day mortality was similar with inappropriate definitive therapy when compared to appropriate definitive therapy in the group of CnSPA (46.0% vs 29.4%, respectively; *p* = 0.085). The result may be affected by the sample size (50 vs 51), different clinical characteristics in the two groups (ie. APACHE II scores, 14.5 vs 13.0, *p* = 0.01) and selection bias. Meanwhile, it may be also explained by the lower effectiveness of non-cephalosporins/carbapenems drugs in the treatment of CnSPA BSI. Further studies should focus on the treatment of CnSPA.

The study has several limitations. First, it was retrospective in nature; therefore, it may be subject to selection bias and treatment options over the 10 entire years may be changeable (ie. dosage and duration of antibiotics, interventions in invasive procedures). Second, as the large number of variables collected and the potential effects of residual confounding, some variables may be significant by chance alone. Third, it was a single center study containing little data; therefore, the results may not be applicable to other hospitals. Indeed, it is essential to evaluate the strains prevalent locally and the condition of the patients before selecting the optimal dosage regimen. In addition, we did not include adverse events, cost, and repeated infections in our analysis of risk factors for mortality.

## Conclusion

We found that pulmonary disease and exposure to carbapenems were independent risk factors for developing CnSPA BSIs. Appropriate therapy and prompt therapy reduced mortality rates. However, combination therapy was no better than monotherapy for reducing mortality associated with PA BSI. Combination therapy with β-lactams was as effective as carbapenems for treatment of PA BSIs.

## Additional files

Additional file 1: **Figure S1.** Flowchart of the case selection process. (PDF 55 kb)

Additional file 2: **Figure S2.** Annual incidence of PA bloodstream infections (PA and CnSPA) in hospital and ICU: 2007–2016. (PDF 40 kb)

Additional file 3: **Figure S3.** Annual incidence of *Pseudomonas aeruginosa* bloodstream infections (PA and CnSPA): 2007–2016. (PDF 92 kb)

Additional file 4: **Table S1.** Risk factors associated with 30 day mortality in patients with CnSPA BSIs. **Table S2.** Risk factors associated with 30 day mortality in patients with CSPA BSIs. **Table S3.** Clinical characteristics, infection status, and outcomes of patients with PA BSI who received definitive therapy. **Table S4.** Microbial susceptibility to piperacillin/tazobactam (PDF 100 kb)

## Abbreviations

APACHE II: Acute physiology and chronic health status scoring system II
BLBLIs: Beta-lactam/beta-lactam inhibitor combinations
BSI: Bloodstream infection
CCI: Charlson Comorbidity Index
CIs: Confidence intervals
CnSPA: Carbapenem-non-susceptible *Pseudomonas aeruginosa*
CSPA: Carbapenem-susceptible *Pseudomonas aeruginosa*
ICU: Intensive care unit
OR: Odds ratio
PA: *Pseudomonas aeruginosa*
