# Supplementary figures and images for: A retrospective analysis of Pseudomonas aeruginosa bloodstream infections: prevalence, risk factors, and outcome in carbapenem-susceptible and -non-susceptible infections
Source: Antimicrob Resist Infect Control. 2019 Apr 25;8:68. doi: 10.1186/s13756-019-0520-8 (PMC6485151; doi:10.1186/s13756-019-0520-8)

Figure S1. Flowchart of the case selection process

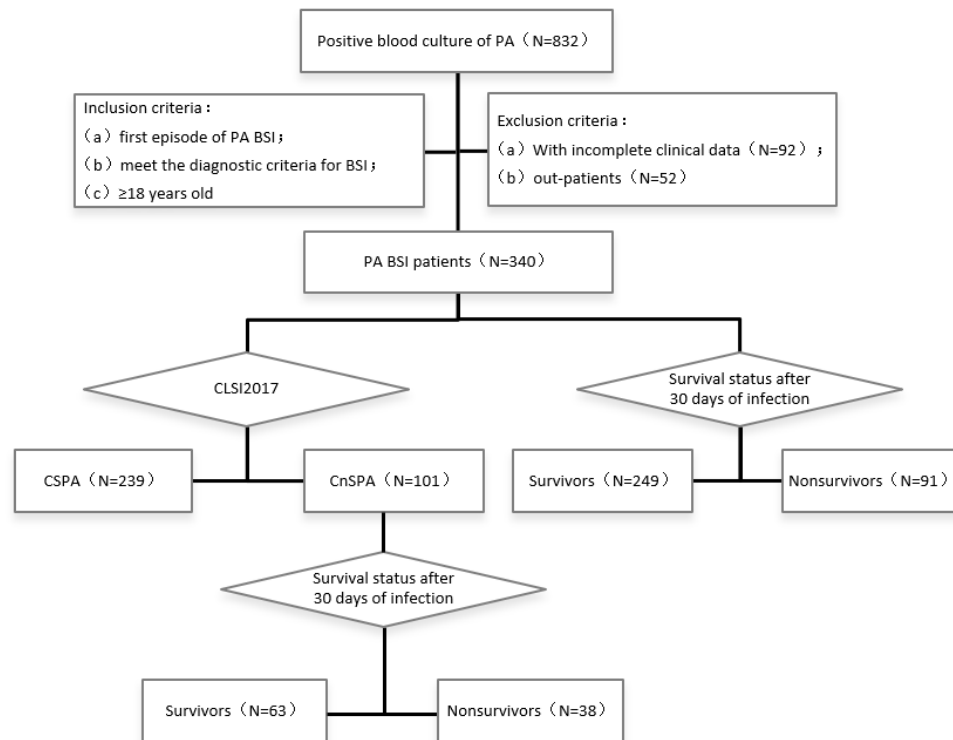

Supplement: Supplementary file 1 — Figure S1. Flowchart of the case selection process. (PDF 55 kb) [file 13756_2019_520_MOESM1_ESM.pdf]

1000 Patient-days

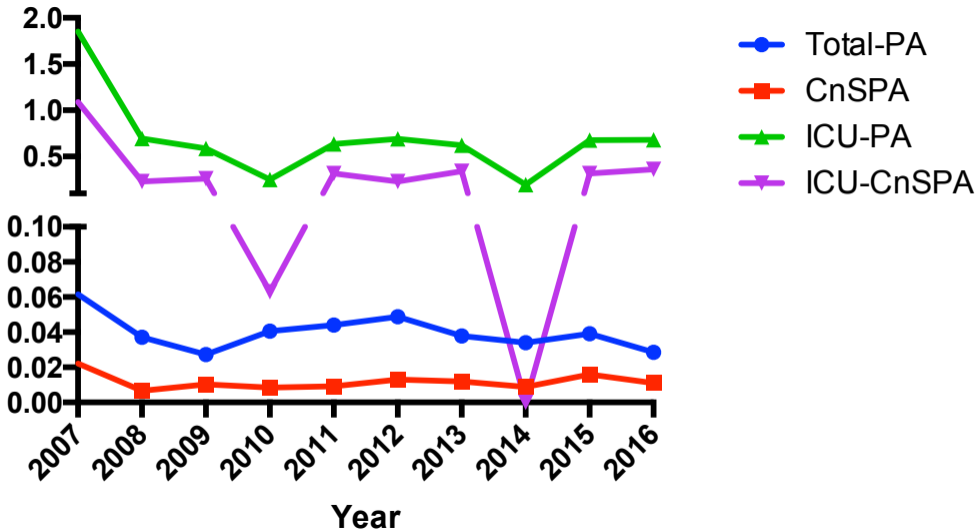

Supplement: Supplementary file 2 — Figure S2. Annual incidence of PA bloodstream infections (PA and CnSPA) in hospital and ICU: 2007–2016. (PDF 40 kb) [file 13756_2019_520_MOESM2_ESM.pdf]

Figure S3. Annual incidence of *Pseudomonas aeruginosa* bloodstream infections (PA and CnSPA):2007-2016

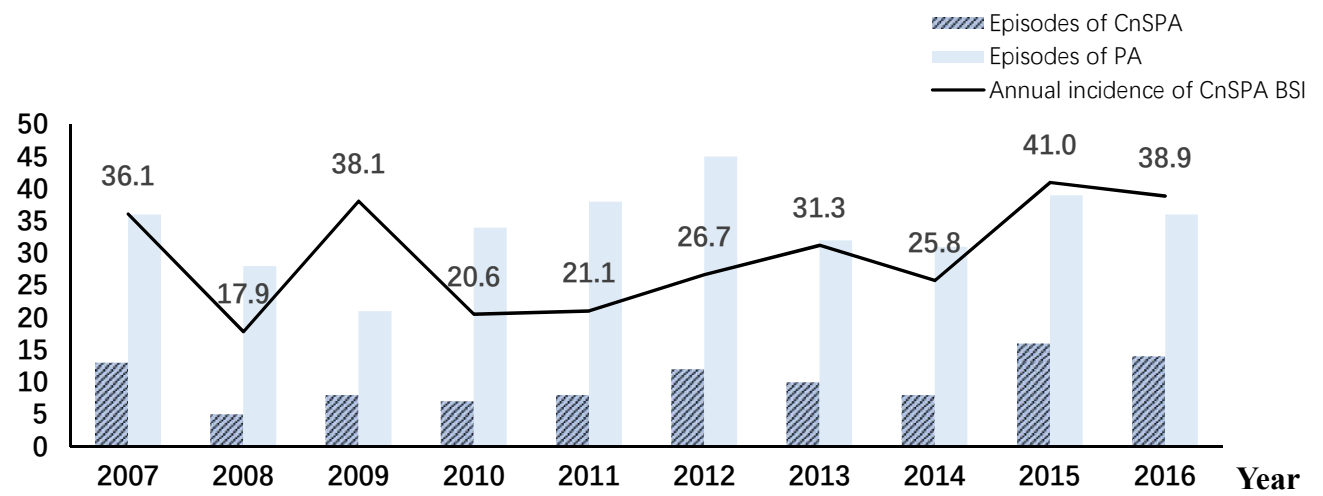

Supplement: Supplementary file 3 — Figure S3. Annual incidence of Pseudomonas aeruginosa bloodstream infections (PA and CnSPA): 2007–2016. (PDF 92 kb) [file 13756_2019_520_MOESM3_ESM.pdf]
