# Supplementary material for: A retrospective analysis of Pseudomonas aeruginosa bloodstream infections: prevalence, risk factors, and outcome in carbapenem-susceptible and -non-susceptible infections
Source: Antimicrob Resist Infect Control. 2019 Apr 25;8:68. doi: 10.1186/s13756-019-0520-8 (PMC6485151; doi:10.1186/s13756-019-0520-8)
Supplement: Supplementary file 4 — Table S1. Risk factors associated with 30 day mortality in patients with CnSPA BSIs. Table S2. Risk factors associated with 30 day mortality in patients with CSPA BSIs. Table S3. Clinical characteristics, infection status, and outcomes of patients with PA BSI who received definitive therapy. Table S4. Microbial susceptibility to piperacillin/tazobactam (PDF 100 kb) [file 13756_2019_520_MOESM4_ESM.pdf]

Table S1. Risk factors associated with 30 day mortality in patients with CnSPA BSIs

	Univariate analysis			Multivariate analysis			
	Survivors	Non-survivors				95% CI for EXP(B)	
	(N=63)	(N=38)	*P* values	Sig.	Exp(B)	Lower	Upper
Demographics							
Male, n (%)	40 (63.5)	28 (73.7)	0.290				
Age, mean ± SD	58.0±18.1	64.2±17.4	0.097				
Department							
ICU	23 (36.5)	26 (68.4)	0.002				
Hematology Department	8 (12.7)	1 (2.6)	0.174				
Hepatobiliary and Pancreatic	15 (23.8)	7 (18.4)	0.525				
Comorbidities and underlying disease							
Diabetes mellitus	4 (6.3)	7 (18.4)	0.119				
Hypertension	12 (19.0)	15 (39.5)	0.025				
Respiratory disease	11 (17.5)	19 (50.0)	0.001				
Solid tumor	12 (19.0)	6 (15.8)	0.679				
Hematological disease	9 (14.3)	1 (2.6)	0.120				
Leukocythemia	3 (4.8)	0 (0)	0.447				
CCI, median [IQR]	1.0 [2.0]	1.5 [1.0]	0.700				
APACHE II score on admission, mean±SD	11.9±5.5	15.1±6.2	0.009				
Before BSI (within 90 days)							
Past hospitalization	5 (7.9)	2 (5.3)	0.914				
ICU stay	34 (54)	28 (73.7)	0.049				
Surgery	24 (38.1)	13 (34.2)	0.695				
Blood transfusion	26 (41.3)	20 (52.6)	0.267				
Corticosteroids	8 (38.1)	13 (68.8)	0.010	0.028	8.055	1.246	52.091
Immunosuppressive drugs	5 (7.9)	2 (5.3)	0.914				
Invasive therapy							
Central venous catheterization	31 (49.2)	30 (78.9)	0.003				
Arterial catheterization	24 (38.1)	26 (68.4)	0.003				
Mechanical ventilation	33 (52.4)	28 (73.7)	0.034				
Endotracheal intubation or incision	17 (27)	18 (47.4)	0.037				
Urinary catheterization	31 (49.2)	26 (68.4)	0.059				
Indwelling gastrointestinal tube	25 (39.7)	26 (68.4)	0.005				
Hemodialysis	4 (6.3)	9 (23.7)	0.027				
Antibiotic exposure							
Third/fourth generation Cephalosporin	7 (11.1)	7 (18.4)	0.303				
Carbapenem	30 (47.6)	19 (50)	0.817				
BLBLI	23 (36.5)	26 (68.4)	0.002				
Aminoglycoside	1 (1.6)	2 (5.3)	0.292				
Polymyxin	0 (0)	1 (2.6)	0.196				

Fosfomycin	2 (3.2)	3 (7.9)	0.289
Fluoroquinolone	7 (11.1)	8 (21.1)	0.173
Likely source of bacteremia			
Respiratory tract	27 (42.9)	26 (68.4)	0.013
Biliary tract	15 (23.8)	7 (18.4)	0.525
Intra-abdominal	9 (14.3)	4 (10.5)	0.810
Urinary tract	1 (1.6)	0	0.330
Unknown source	6 (9.5)	2 (5.3)	0.698
Infection status			
Neutropenia	9 (14.3)	4 (10.5)	0.810
Hypoalbuminemia	11 (18.6)	15 (45.5)	0.006
APACHE II score, mean± SD	13.9±4.8	16.2±5.9	0.036
Pitt score, mean± SD	1.8±1.7	2.6±2.3	0.085
After BSI			
Corticosteroids	34 (54.0)	20 (52.6)	0.063
Antibiotic within 30 days after BSI			
Third/fourth generation			
Cephalosporin	15 (23.8)	5 (13.2)	0.193
BLBLI	39 (61.9)	21 (55.3)	0.510
Aminoglycoside	4 (6.3)	3 (7.9)	1.000
Carbapenem	40 (63.5)	20 (52.6)	0.282
Polymyxin	1 (1.6)	1 (2.6)	1.000
Fosfomycin	6 (9.5)	2 (5.3)	0.698
Fluoroquinolone	27 (42.9)	12 (31.6)	0.259
Empirical therapy	54 (85.7)	27 (71.1)	0.073
Monotherapy	45 (71.4)	20 (52.6)	0.324
Combination therapy	9 (14.3)	7 (18.4)	0.324
Containing carbapenem	2 (3.2)	3 (7.9)	0.558
Containing BLBLI	4 (6.3)	4 (10.5)	0.709
Appropriate therapy	18 (28.6)	12 (31.6)	0.749
Active drug against PA			
0	42 (66.7)	27 (71.1)	0.646
1	16 (25.4)	10 (26.3)	0.918
2	4 (6.3)	1 (2.6)	0.718
≥3	1 (1.6)	0	0.330
Definitive therapy	57 (90.5)	23 (60.5)	<0.001
Third/fourth generation			
Cephalosporin	15 (23.8)	5 (13.2)	0.193
Ceftazidime	2 (3.2)	1 (2.6)	0.875
Cefepime	9 (14.3)	3 (7.9)	0.519
BLBLI	23 (36.5)	9 (23.7)	0.180
Aminoglycoside	6 (9.5)	1 (2.6)	0.359
Carbapenem	26 (41.3)	11 (28.9)	0.213

Polymyxin	2 (3.2)	0	0.167				
Fosfomycin	3 (4.8)	3 (7.9)	0.833				
Fluoroquinolone	23 (36.5)	9 (23.7)	0.180				
Antifungal agent	29 (46.0)	11 (28.9)	0.089				
Definitive monotherapy	39 (61.9)	16 (42.1)	0.920				
Definitive Combination therapy	18 (28.6)	7 (18.4)	0.920				
Containing carbapenem	6 (9.5)	3 (7.9)	1.000				
Containing BLBLI	6 (9.5)	2 (5.3)	0.698				
Definitive inappropriate therapy	27 (42.9)	23 (60.5)	0.085				
Definitive appropriate therapy	36 (57.1)	15 (39.5)	0.085				
Active drug against PA							
1	29 (46.0)	12 (31.6)	0.152				
BLBLI	13 (20.6)	6 (15.8)	0.546				
Carbapenem	11 (17.5)	5 (13.2)	0.566				
2	5 (7.9)	3 (7.9)	1.000				
3	2 (3.2)	0	0.167				
Delay time from blood culture results to appropriate therapy							
≤24 h	27 (42.9)	7 (18.4)	0.012	0.022	0.108	0.016	0.723
24–48 h	2 (3.2)	3 (7.9)	0.558				
>48 h	8 (12.7)	5 (13.2)	1.000				

Data are expressed as numbers (%) unless otherwise stated; Abbreviations: PA: Pseudomonas aeruginosa; CnSPA: carbapenem-non-susceptible PA; BSI: bloodstream infection; LOS: length of stay; ICU: intensive care unit; CCI, Charlson comorbidity index; BLBLI： Beta-lactam/beta-lactam inhibitor combination

Table S2. Risk factors associated with 30 day mortality in patients with CSPA BSIs

	Univariate analysis			Multivariate analysis			
	Survivors	Non-survivors				95% CI for EXP(B)	
	(N=186)	(N=53)	*P* values	Sig.	Exp(B)	Lower	Upper
Empirical therapy							
Monotherapy	142 (87.1)	37 (92.5)	0.502				
Combination therapy	21 (12.9)	3 (7.5)	0.502				
Appropriate therapy	163 (87.6)	40 (75.5)	0.029				
Active drug against PA							
0	23 (12.4)	13 (24.5)	0.029				
1	146 (78.5)	37 (69.8)	0.188				
≥2	17 (9.1)	3 (5.7)	0.599				
Definitive therapy							
Third/fourth generation							
Cephalosporin	25 (13.4)	4 (7.5)	0.246				
Ceftazidime	2 (1.1)	1 (1.9)	0.656				
Cefepime	10 (5.4)	1 (1.9)	0.485				
BLBLI	81 (43.5)	14 (26.4)	0.025				
Aminoglycoside	10 (5.4)	3 (5.7)	1.000				
Carbapenem	80 (43.0)	20 (37.7)	0.492				
Polymyxin	0	1 (1.9)	0.082				
Fosfomycin	6 (3.2)	2 (3.8)	1.000				
Fluoroquinolone	43 (23.1)	10 (18.9)	0.511				
Antifungal agent	45 (24.2)	24 (45.3)	0.003	<0.001	3.664	1.799	7.462
Definitive therapy							
Monotherapy	138 (83.6)	28 (84.8)	0.863				
BLBLI	63 (33.9)	12 (22.6)	0.120				
Carbapenem	67 (36.0)	16 (30.2)	0.431				
Combination therapy	27 (16.4)	5 (15.2)	0.863				
Appropriate therapy	161 (86.6)	32 (42.8)	<0.001	<0.001	0.168	0.080	0.352
Active drug against PA							
0	25 (13.4)	21 (39.6)	<0.001				
1	133 (71.5)	26 (49.1)	0.002				
≥2	28 (15.1)	6 (11.3)	0.493				

Data are expressed as numbers (%) unless otherwise stated; Abbreviations: CSPA: carbapenem-non-susceptible Pseudomonas aeruginosa; BSI: bloodstream infection; BLBLI: Beta-lactam/beta-lactam inhibitor combination

Table S3. Clinical characteristics, infection status, and outcomes of patients with PA BSI who received definitive therapy

Parameters	Monotherapy (N=221)	Combination therapy (N=57)	*P*
APACHE II scores at admission, mean± SD	12.0±5.3	11.8±5.1	0.814
CCI, median [IQR]	2.0 [1.0]	2.0 [1.5]	0.309
Infection status			
Neutropenia	49 (22.2)	10 (17.5)	0.446
Hypoalbuminemia	71 (32.1)	19 (33.3)	0.862
APACHE II scores, mean± SD	15.2±4.7	14.6±5.1	0.420
PITT scores, median [IQR]	1.0 [1.0]	1.0 [1.0]	0.784
Infectious source: respiratory tract	61 (27.6)	18 (31.6)	0.553
Non-survivors	44 (19.9)	12 (21.5)	0.848

Data are expressed as numbers (%) unless otherwise stated; Abbreviations: PA: Pseudomonas aeruginosa; BSI: bloodstream infection; CCI, Charlson comorbidity index

Table S4. Microbial susceptibility to piperacillin/tazobactam

	PA (N=340)	CSPA (N=239)	CnSPA (N=101)
Susceptible, n (%)	268 (78.8)	216 (90.4)	52 (51.5)
Intermediate, n (%)	23 (6.7)	11 (4.6)	12 (11.9)
Resistance, n (%)	49 (14.4)	12 (5.0)	37 (36.6)

Abbreviations: PA: Pseudomonas aeruginosa; CSPA: carbapenem-susceptible PA; CnSPA: carbapenem-non-susceptible PA
